# Improvement of chicken genome editing efficiency in vitro using ribonucleoprotein-mediated CRISPR/Cas9 delivery

**DOI:** 10.1186/s40104-026-01402-1

**Published:** 2026-05-04

**Authors:** Yujin Han, Chan Young Kwon, Jae Yong Han

**Affiliations:** 1 Major in Biomodulation, Animal Science and Biotechnology, Department of Agricultural Biotechnology and Research Institute of Agriculture and Life Sciences, College of Agriculture and Life Sciences, Seoul National University, Seoul, Republic of Korea; 2 Avinnogen Co., Ltd., Seoul, Republic of Korea

**Keywords:** Chicken, CRISPR/Cas9, Genome editing, Indel profiling, Ribonucleoprotein (RNP)

## Abstract

**Background:**

Limitations in genome editing of avian species, including chicken, due to the inaccessibility of one-cell zygotes, have led to the manipulation of primordial germ cells (PGCs) as the primary approach for generating genetically engineered birds. Although plasmid-mediated delivery of clustered regularly interspaced short palindromic repeats/CRISPR-associated protein 9 (CRISPR/Cas9) has been widely used, it has several limitations, including delayed nuclease activation, increased off-target effects, cytotoxicity, and the inability to apply in vivo selection strategies. Collectively, these limitations highlight the need to develop strategies that achieve high on-target activity at the initial editing step.

**Results:**

In this study, we compared plasmid- and ribonucleoprotein (RNP)-mediated CRISPR/Cas9 delivery in Leghorn male hepatoma (LMH) cells and in PGCs targeting three loci: deleted in azoospermia like (*DAZL*), chicken vasa homologue (*CVH*), and stimulated by retinoic acid 8 (*STRA8*). RNP delivery showed comparable or higher cell viability and editing efficiency than plasmid delivery in both cell types. Insertion/deletion (indel) profiling revealed broader and more diverse mutation patterns with RNPs, consistent with a shift in repair pathway engagement toward microhomology-mediated end joining (MMEJ), which favors larger deletions. Off-target analysis further showed substantially reduced off-target editing and high specificity with RNP delivery.

**Conclusions:**

Together, these findings demonstrate that RNP delivery improves efficiency, reduces cytotoxicity, and improves precision, providing a more reliable platform for chicken genetic engineering.

**Supplementary Information:**

The online version contains supplementary material available at 10.1186/s40104-026-01402-1.

## Background

Chicken (*Gallus gallus*) is a major global food source, providing essential protein to a rapidly growing human population. Beyond agriculture, the chicken is also a valuable model organism in biotechnology and immunology due to its distinctive developmental and reproductive physiology [1]. Genome editing has been increasingly explored in chickens to improve disease resistance and production efficiency [2], underscoring its potential to address economic and public health challenges. Given the need to enhance production traits, improve disease resistance, and accommodate changing environmental conditions, advanced genetic modification technologies are required in poultry science [3].

In mammalian systems, direct microinjection of genome editing tools into one-cell-stage zygotes enables the efficient generation of genetically modified animals [4]. However, this method is not feasible in avian systems because their one-cell-stage zygotes contain an opaque yolk, and oviposition occurs after the embryo has reached the Eyal-Giladi and Kochav (EGK) stage X, which complicates manipulation [5]. Accordingly, methods based on primordial germ cells (PGCs), the germline progenitors that transmit genetic modifications to offspring, are widely used to generate genetically-modified chickens [6, 7].

Programmable genome editing tools, including zinc finger nucleases (ZFNs), transcription activator-like effector nucleases (TALENs), and clustered regularly interspaced short palindromic repeats/CRISPR-associated protein (CRISPR/Cas) system, have enabled efficient and precise modification of desired genes across diverse organisms [8, 9]. Among these, the CRISPR/Cas9 system is the most widely adopted platform due to its simplicity, efficiency, and flexibility, requiring only guide RNA (gRNA) redesign to target new loci [10].

Traditionally, genome editing in PGCs has relied predominantly on plasmid delivery systems for CRISPR/Cas9 (i.e., plasmids encoding CRISPR/Cas9 and single-guide RNA (sgRNA)). However, plasmid delivery requires multiple intracellular steps, including transcription and translation [11, 12], thereby delaying nuclease activity and reducing temporal precision. Sustained CRISPR/Cas9 expression may also increase off-target editing activity [13]. Furthermore, plasmid delivery typically exhibits low editing efficiency in germline-related cells, including chicken PGCs [14]. Due to these low efficiencies, antibiotic selection is frequently used to enrich successfully transfected cells. However, because selection cannot be readily applied in vivo, plasmid delivery faces substantial practical limitations.

**Fig. 1:**
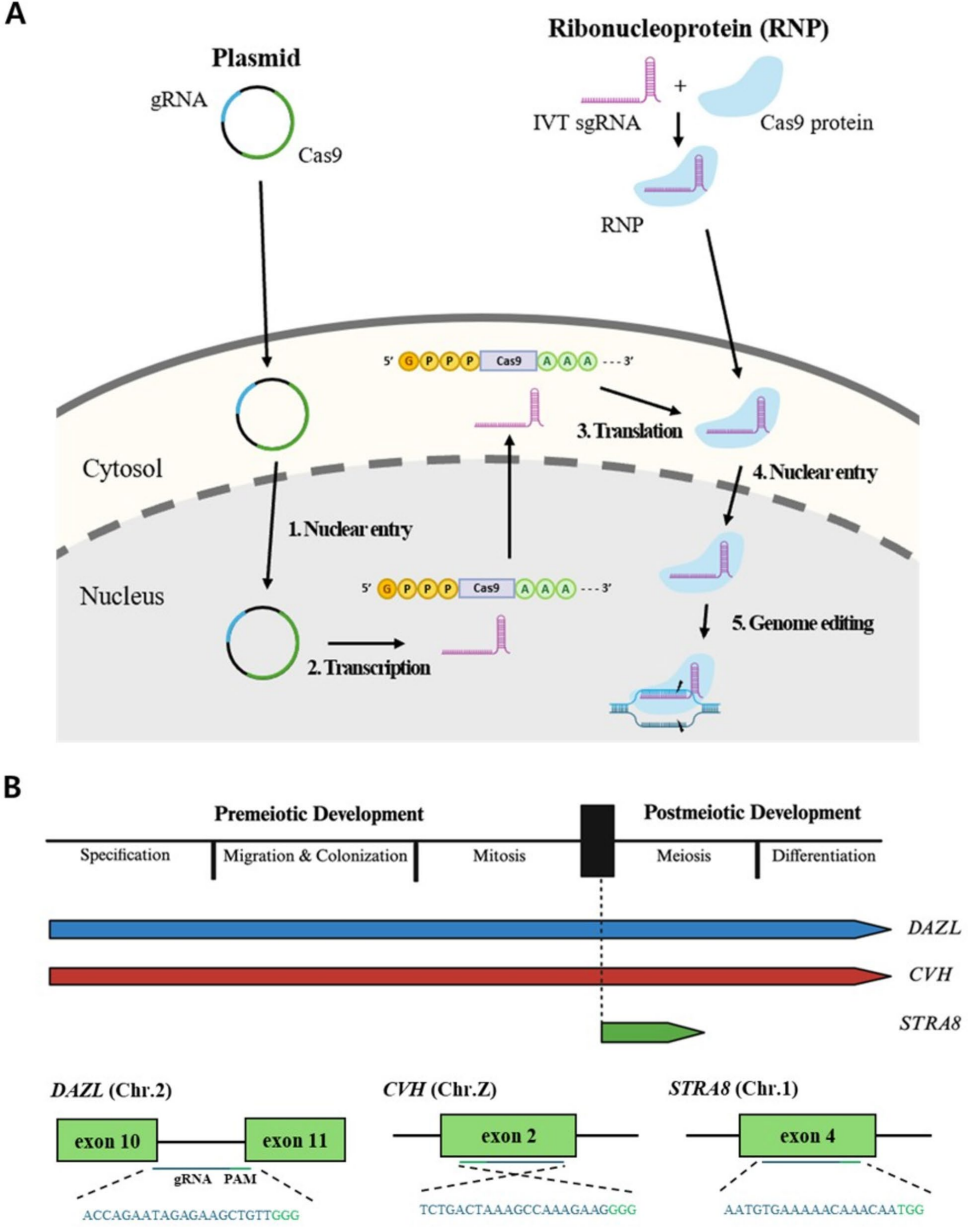
Schematics of CRISPR editing workflows, germ-cell development markers, and gRNA targeting. **A** Schematic overview of plasmid- and RNP-mediated CRISPR genome editing workflows. **B** Schematic representation of target genes. Developmental timeline of germ cells illustrating the expression windows of *DAZL*, *CVH*, and *STRA8* across pre-meiotic and post-meiotic stages. Schematic representation of the gRNAs designed for each gene, indicating their target sites within the corresponding exons. Green boxes denote targeted exons, while blue and green lines highlight the gRNA target sequences and PAM (5′–NGG–3′), respectively

As an alternative, ribonucleoprotein (RNP; i.e., pre-assembled CRISPR/Cas9 protein complexed with sgRNA) delivery has emerged as a promising strategy [15] (Fig. 1A). RNPs can induce immediate and robust editing after delivery, reduce off-target activity owing to their transient activity, and frequently impose less cellular stress [16]. Although numerous studies in mammalian systems have reported enhanced editing efficiencies and reduced cytotoxicity with RNP delivery compared with plasmids [17–19], systematic comparative analyses in avian species remain limited [20].

Here, we evaluate the feasibility of CRISPR/Cas9 RNP delivery for chicken genome editing by systematically comparing editing efficiency, cell viability, and off-target activity between plasmid and RNP delivery in two chicken cell types: Leghorn male hepatoma (LMH) cells as a representative somatic cell line and PGCs as germline-competent cells used for germline transmission. We target three germline-related genes that regulate germ cell specification, maintenance, and identity: deleted in azoospermia like (*DAZL*), chicken vasa homologue (*CVH*), and stimulated by retinoic acid 8 (*STRA8*) (Fig. 1B). We further investigated whether the delivery method alters the engagement of the DNA repair pathway. Collectively, our findings develop a framework for more reliable chicken genome editing and inform future in vivo system applications.

## Methods

### Vector construction

gRNAs targeting *DAZL* and *STRA8* were designed based on sequences validated in previous literature [21, 22]. gRNA targeting *CVH* was designed in Geneious Prime (Biomatters Ltd., Auckland, New Zealand) (Table 1) using the reference chicken genome (Gallus_gallus-6.0, GRCg6a) available at the National Center for Biotechnology Information (NCBI).

**Table 1:** Target site information for CRISPR/Cas9 genome editing in chicken cells

Gene	**5′**–**gRNA Seq**–**3′**	**Chromosome**	**Locus**
*DAZL*	ACC AGA ATA GAG AAG CTG TT	CM000094.5	34,430,607
*CVH*	TCT GAC TAA AGC CAA AGA AG	CM000122.5	16,931,199
*STRA8*	AAT GTG AAA AAC AAA CAA	CM000093.5	47,900,316

For CRISPR/Cas9 plasmid-based genome editing, the plasmid pSpCas9(BB)-2A-Puro (PX459) (#48139, Addgene, MA, USA) was used. gRNA sequences were inserted into the vector by Golden Gate assembly with the BpiI restriction enzyme (#ER1011, Thermo Fisher, MA, USA) and T4 ligase (#2011A, Takara, Shiga, Japan). The corresponding oligonucleotides were synthesized by Cosmogenetech (Seoul, South Korea) (Table S1). The plasmid pSpCas9(BB)-2A-GFP (PX458) (#48138, Addgene) was also used to assess plasmid delivery efficiency (Fig. S1A).

All plasmids were purified using either a Plasmid Transfection-grade Prep Kit (#740490, MACHEREY-NAGEL, Düren, Germany) or an Endo-free plasmid maxi kit (#12362, Qiagen, Hilden, Germany), according to the manufacturer’s instructions**.**

### sgRNA in vitro transcription

sgRNAs targeting *DAZL, CVH,* and *STRA8* were in vitro transcribed (IVT) using the Precision gRNA Synthesis kit (#A29377, Thermo Fisher) according to the manufacturer’s protocol with the corresponding oligonucleotides (Table S1). The quality of the IVT sgRNAs was assessed by electrophoresis on a 2% agarose gel before downstream applications (Fig. S2).

### Cell culture

LMH cells (CRL-2117, American Type Culture Collection (ATCC), VA, USA) were sub-passaged every 2–4 d in Waymouth's Medium (#11220035, Thermo Fisher) supplemented with 10% of fetal bovine serum (FBS) (#SH30919.03, Cytiva, MA, USA) and 1% of Antibiotic–Antimycotic (ABAM) (#15240062, Thermo Fisher). LMHs were incubated at 37 °C in a 5% CO_2_ atmosphere and 60%–70% relative humidity.

Cultured chicken PGCs were sub-passaged every 3–5 d in PGC culture medium [23]. PGCs were incubated at 37 °C in a 5% CO_2_ atmosphere and 60%–70% relative humidity.

### Transfection

Because the mechanisms of plasmid and RNP delivery differ fundamentally, strict mass- or molar-equivalent matching does not necessarily yield comparable active nuclease exposure. Accordingly, we used the conditions specified in previously validated protocols for each format [23] and manufacturers’ recommendations. These conditions were selected to compare practical editing performance under optimized use conditions rather than equimolar comparisons.

For LMH cells, 3 µL of Lipofectamine 2000 reagent (#11668027, Thermo Fisher) and 2 µg of CRISPR plasmid were mixed in 500 µL of Opti-MEM (#31985070, Thermo Fisher), and the mixture was applied to cells. LMH cells were seeded at a density of 1 × 10^5^ cells per well. At the time of transfection, cells were approximately 60%–70% confluent. The medium was replaced with fresh culture medium 6 h after transfection. For PGCs, 3 µL Lipofectamine 2000 reagent and 2 µg of CRISPR plasmid were suspended in 100 µL of Opti-MEM, and the mixture was applied to 1 × 10^5^ cultured PGCs together with 400 µL of serum-free PGC culture medium. Gentle pipetting was performed every 1–1.5 h. The medium was replaced with fresh culture medium 6 h after transfection.

In parallel, for both LMH cells and PGCs, 3 µL of CRISPRMAX reagent (#CMAX00003, Thermo Fisher), 2.5 µL of Cas PLUS reagent, 1.25 µg of TrueCut™ Cas9 Protein v2 (#A36498, Thermo Fisher), and 250 ng of IVT sgRNAs were suspended in 100 µL Opti-MEM, and this mixture was applied to 1 × 10^5^ cells with 400 µL culture medium. RNPs were prepared by pre-incubating Cas9 protein with IVT sgRNA at a 1:1 molar ratio as recommended by the manufacturer. For PGCs, gentle pipetting was carried out at 1–1.5 h intervals for the first 6 h after transfection.

Preliminary optimization of RNP transfection conditions was performed in LMH cells to determine robust RNP input, complex formation, and transfection parameters before application in PGCs (Fig. S3).

### gDNA extraction, PCR, and PCR purification

Cells were harvested 48 h after transfection, and genomic DNA (gDNA) was extracted from each cell sample. The gDNA concentration was measured using a NanoDrop-2000 (Thermo Scientific). The final gDNA concentration for each sample was diluted to 100 ng/µL and used for PCR. Primers were designed using Geneious Prime according to the reference chicken genome and synthesized (Cosmogenetech) (Table S2).

PCR reactions were performed with the total PCR mixture volume of 20 µL containing 0.4 µL of dNTP (10 mmol/L each) (#DN112-10h, Biofact, Seoul, South Korea), 2 µL of 10X Buffer, 0.1 µL of Taq Polymerase (5 units/µL) (#ST111-50h, Biofact), 4 µL of 5X Band Helper (#BB741-10k, Biofact), 1 µL of each primer (10 μmol/L each), 2 µL of extracted gDNA, and 9.5 µL of Ultra-Pure Water (UPW) (#ML019-02, Welgene, Namcheon, South Korea). PCR was conducted under the following thermocycling conditions: initial denaturation at 95 °C for 5 min, followed by 35 cycles of denaturation at 95 °C for 30 s, annealing at the appropriate temperature (Table S2) for 30 s, extension at 72 °C for 1 min, then a final extension at 72 °C for 10 min. After confirming successful PCR amplification of the target sites on a 1.2% agarose gel via gel electrophoresis, each amplicon was purified using the Wizard™ SV Gel and PCR Clean-Up System (#A9282, Promega, WI, USA).

### Next-generation sequencing analysis

Next-generation sequencing (NGS) was outsourced to Bionics (Seoul, South Korea) using the MiSeq system (Illumina, CA, USA). Each amplicon was analyzed at approximately 30,000 reads. The resulting FASTQ (.fastq.gz) files were aligned and analyzed using CRISPResso2 [24]. The quantification window center was set to 3 bp upstream of the protospacer adjacent motif (PAM), and the window size was defined as ± 10 bp around the center. Only insertion/deletion (indel) frequencies were quantified, whereas substitutions were excluded.

The Microhomology-Predictor tool [25] was used to predict microhomology-mediated end joining (MMEJ)-associated editing patterns (Table S3). MMEJ-associated editing was defined as deletion events whose junctions contained ≥ 2-bp microhomology, consistent with patterns predicted by Microhomology-Predictor. For analyses focusing on MMEJ-associated repair, an expanded quantification window of ± 20 bp around the center was applied to ensure accurate capture of larger deletion events.

### Prediction and analysis of off-target sites

The Cas-OFFinder tool [26] was used to predict ten putative off-target sites for each gRNA in silico. Potential off-target sites with up to four mismatches or a single DNA bulge in the chicken reference genome were identified (Table S4).

Each site was amplified with target-specific primers (Table S2) and analyzed using Tracking of Indels by Decomposition (TIDE) [27] on an ABI Prism 3730XL DNA Analyzer (Thermo Fisher) and available software (tide.nki.nl). When no off-target editing activities were detected, the off-target frequency was set to the TIDE limit of detection (0.1%) as a conservative substitute to enable calculation of the specificity ratio.

Amplicons were also analyzed via T7 endonuclease I (T7E1) assay (#M0302L, New England Biolabs, MA, USA). Following denaturation, the amplicons were reannealed to form heteroduplex DNA. Subsequently, the heteroduplex amplicons were treated with T7E1 for 20 min at 37 °C and then analyzed by 2% agarose gel electrophoresis. The image was taken and analyzed by the ChemiDoc XRS + System (Bio-Rad, CA, USA) and Image Lab Software (Bio-Rad).

### Statistical analysis

Statistical analysis was performed using Microsoft Excel (Microsoft, NM, USA). Significant differences among the groups were evaluated by Student’s *t*-test. A value of *P* < 0.05 indicated statistical significance. All data are presented as mean ± standard error of the mean (SEM) from at least three independent experiments.

## Results

### Enhanced cellular viability and genome editing efficiency by RNP in LMH cells

**Fig. 2:**
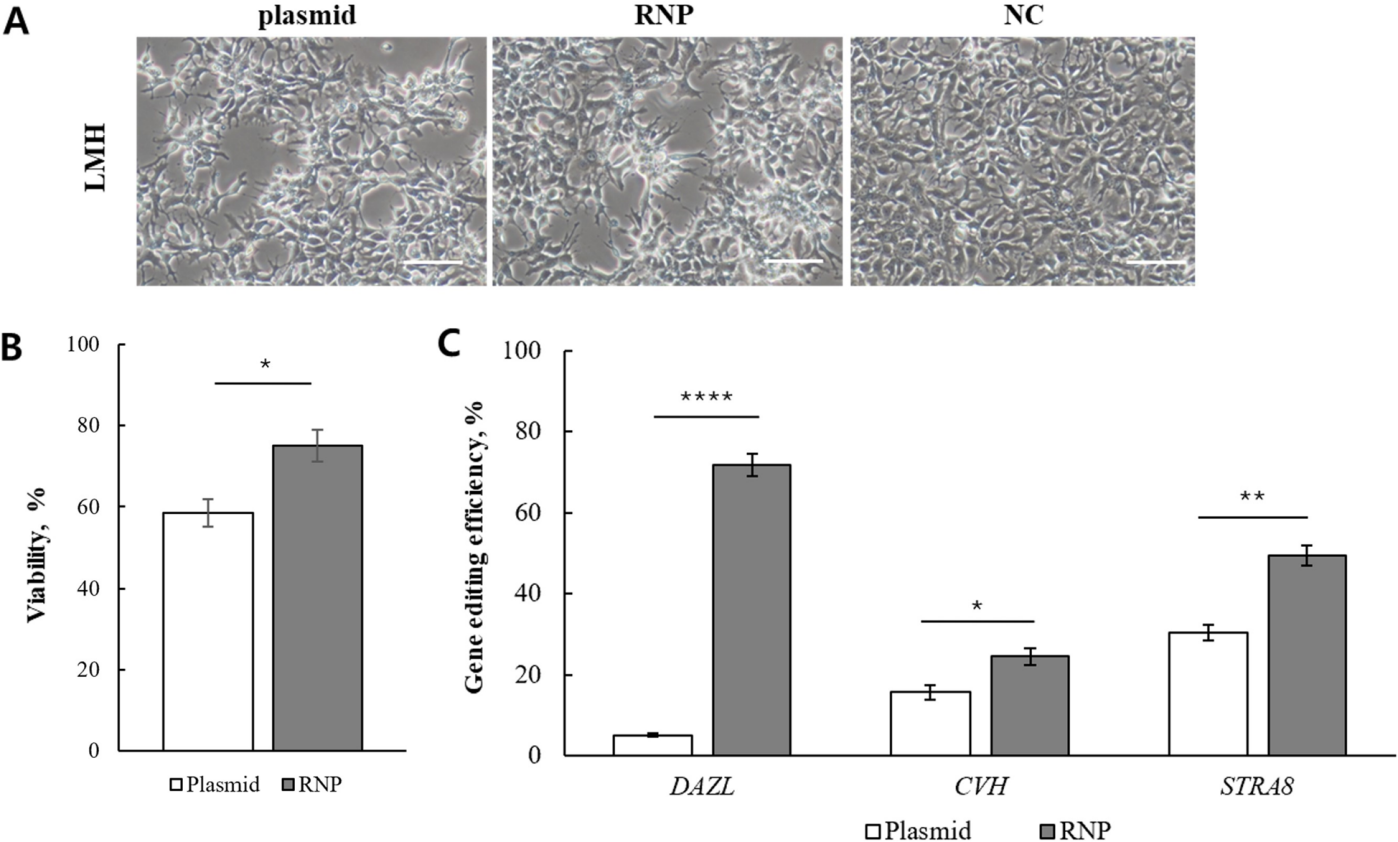
Comparative analysis of viability and editing efficiency between RNP and plasmid delivery in LMH cells. **A** Representative bright-field images of LMH cells 48 h after transfection with plasmid or RNP. Images show overall cell morphology and viability following transfection. Scale bar = 50 µm. **B** Quantification of LMH cell viability 48 h after transfection. Cell viability was assessed by staining cells with trypan blue and manual counting. RNP delivery maintained markedly higher cell viability compared with plasmid delivery. **C** Genome editing efficiencies without selection for *DAZL*, *CVH,* and *STRA8* loci measured by NGS. RNP delivery resulted in significantly higher editing efficiency than plasmid transfection. Data are presented as mean ± SEM (*n* = 3); Student’s *t*-test; *P < 0.05, **P < 0.01, and ****P < 0.0001

To evaluate the impact of delivery methods on cell viability and genome editing efficiency in LMH cells, we compared RNP delivery with plasmid delivery. Bright-field microscopy images captured 48 h post-transfection revealed that both groups maintained healthy morphology, with no apparent cytotoxic effects specific to either delivery method (Fig. 2A). Consistent with these observations, quantitative analysis using trypan blue staining confirmed that RNP delivery preserved significantly higher cell viability (75.02% ± 3.87%) than plasmid delivery (58.47% ± 3.43%) (*P* < 0.05; Fig. 2B). For plasmid delivery, maximal expression efficiency (GFP-positive; 60.73% ± 0.54%) was observed at 48 h post-transfection (Fig. S1B).

Next, we assessed genome editing efficiency at three target sites (Table 1) using NGS. RNP-mediated editing efficiency was significantly higher than plasmid-mediated editing efficiency across all loci in LMH cells (Fig. 2C; Fig. S4A). The most pronounced difference was observed at the *DAZL* locus, where RNPs achieved 71.82% ± 2.76% editing, compared with 5.13% ± 0.30% using plasmids (*P* < 0.0001). Significant improvements were also detected at the *CVH* (24.46% ± 2.04% vs. 15.63% ± 1.76%) (*P* < 0.05) and *STRA8* loci (49.50% ± 2.50% vs. 30.36% ± 1.93%) (*P* < 0.01).

### Enhanced genome editing efficiency by RNP in PGCs

**Fig. 3:**
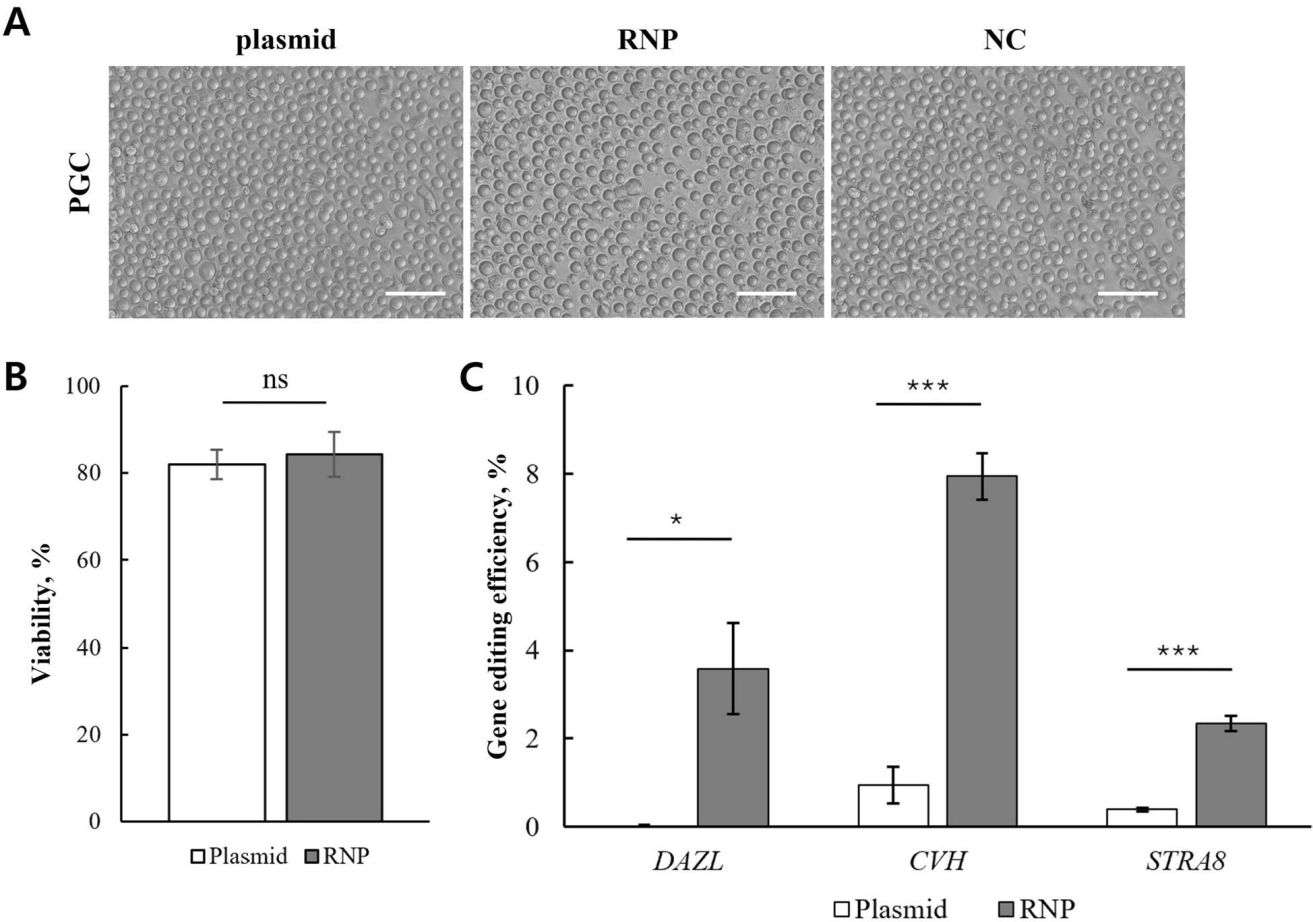
Comparative analysis of viability and editing efficiency between RNP and plasmid delivery in PGCs. **A** Representative bright-field images of PGCs 48 h after transfection with plasmid or RNP. Images show overall cell morphology and viability following transfection. Scale bar = 50 µm. **B** Quantification of PGC viability 48 h after transfection. Cell viability was assessed by staining cells with trypan blue and manual counting. RNP delivery maintained comparable cell viability compared with plasmid delivery. **C** Genome editing efficiencies without selection for *DAZL, CVH*, and *STRA8* loci measured by NGS. RNP delivery resulted in significantly higher editing efficiency than plasmid transfection. Data are presented as mean ± SEM (*n* = 3); Student’s *t*-test; *P < 0.05, and ***P < 0.001

To further assess the efficiency of RNP-mediated genome editing in germline-related cells, we compared RNP and plasmid delivery in PGCs. Bright-field microscopy images captured 48 h post-transfection revealed that both groups maintained similar healthy morphology and cellular integrity, with no apparent cytotoxic effects specific to either delivery method (Fig. 3A). Consistent with these observations, trypan blue staining demonstrated that RNP delivery sustained viability levels comparable to plasmid delivery, with no statistically significant difference (84.27% ± 5.17% vs. 81.95% ± 2.26%) (Fig. 3B). For plasmid delivery, maximal expression efficiency (GFP-positive; 28.73% ± 0.67%) was observed at 48 h post-transfection (Fig. S1B).

Despite similar viability outcomes, genome editing analysis revealed substantial differences between the two delivery approaches. NGS revealed that RNP-mediated editing produced markedly higher editing efficiencies across all three target sites—*DAZL, CVH*, and *STRA8*—when compared with plasmid-mediated editing (Fig. 3C). The most pronounced difference was observed at the *CVH* locus, where RNPs achieved 7.94% ± 0.53% editing, compared with only 0.94% ± 0.42% using plasmids (*P* < 0.001). Similarly, genome editing efficiency at *DAZL* (3.58% ± 1.03% vs. 0.02% ± 0.02%; *P* < 0.05) and *STRA8* loci (2.34% ± 0.18% vs. 0.39% ± 0.04%; *P* < 0.001) was significantly higher in the RNP-treated cells.

### Distinct indel profiles with RNP in LMH cells

**Fig. 4:**
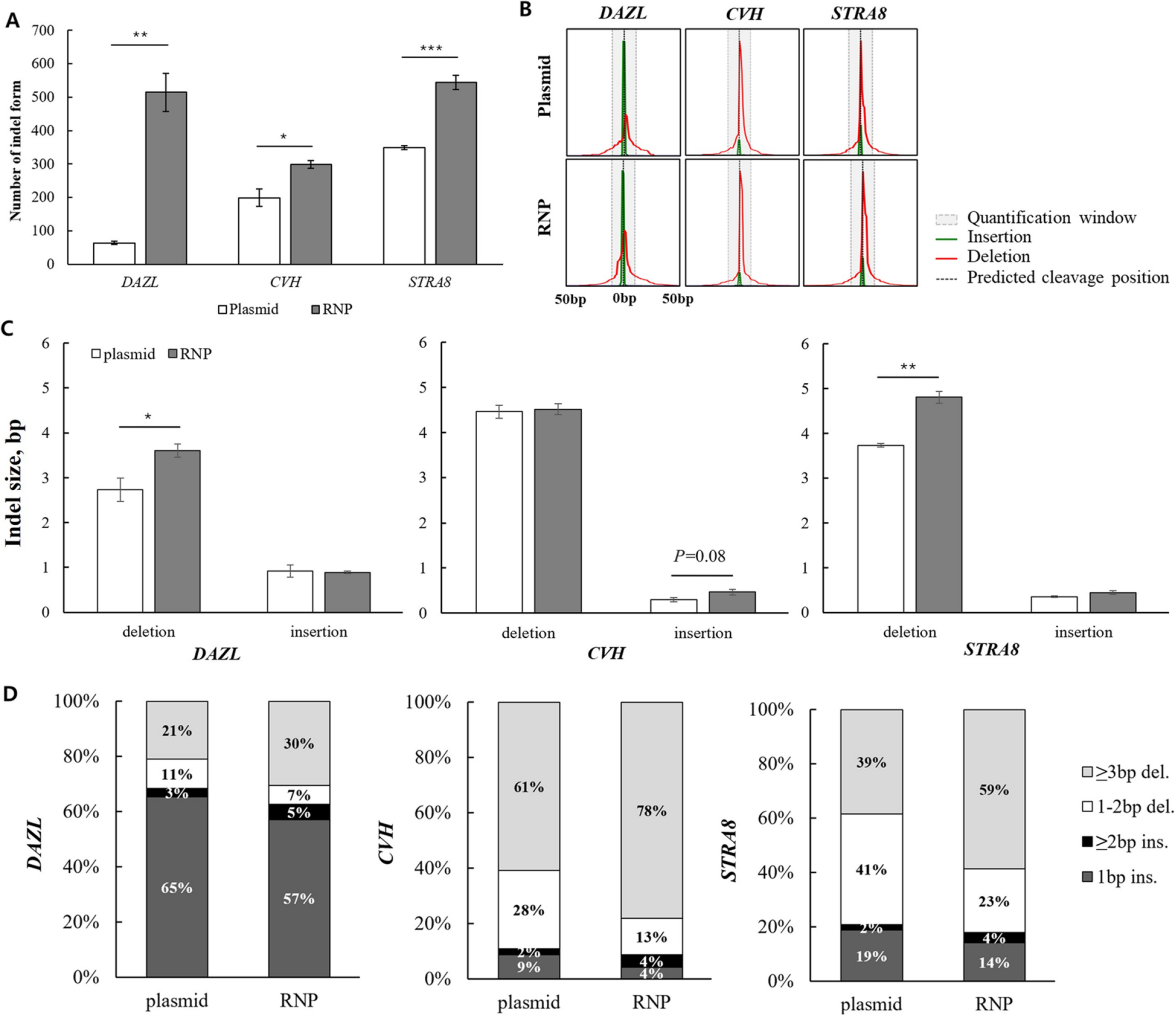
Comprehensive characterization of indel profiles generated by RNP- and plasmid-mediated editing in LMH cells.** A** Quantification of indel diversity generated by RNP and plasmid. For all three target sites, RNP delivery resulted in a significantly greater number of distinct indel patterns than plasmid delivery. **B** Indel position distribution plots. Red peaks represent deletion events and green peaks indicate insertion events. The vertical dashed lines mark the predicted cleavage positions, and the grey-shaded region denotes the quantification window used for analysis. Representative indel plots show that RNP-mediated editing produces broader deletion profiles than plasmid delivery. **C** Comparison of indel sizes between RNP- and plasmid-mediated editing. RNP-mediated editing showed a trend toward generating larger deletion and insertion sizes (bp), with statistically significant differences for *DAZL* and *STRA8.* A similar trend was observed for *CVH*, with insertion size showing a marginal difference. **D** Stacked bar graphs showing the relative proportions of each indel category. Distribution of indel types among total genome editing events, categorized by indel size. For all three target sites, RNP-mediated editing resulted in a significantly higher percentage of ≥ 3 bp deletions than plasmid-mediated editing. Data are presented as mean ± SEM (*n* = 3); Student’s *t*-test; *P < 0.05, **P < 0.01, and ***P < 0.001

To assess whether the delivery method influences the indel pattern of genome editing outcomes, we compared indel profiles generated by RNP- and plasmid-mediated editing in LMH cells. Analysis of indel profiles revealed that RNP delivery consistently produced a markedly greater number of distinct indel patterns detected per sample across all three target sites (Fig. 4A). Specifically, RNP-mediated editing generated significantly higher indel diversity at *DAZL* (514.67 ± 57.01 vs. 63.67 ± 4.91; *P* < 0.01), *CVH* (298.67 ± 11.32 vs. 199.33 ± 25.21; *P* < 0.05), and *STRA8* (544.00 ± 20.50 vs. 349.00 ± 5.29; *P* < 0.001) compared with plasmid-mediated editing.

Indel distribution plots further illustrated the distinct editing patterns generated by the two delivery methods (Fig. 4B). In RNP-mediated edited samples, deletion events (red peaks) were more widely distributed around the predicted cleavage site, producing broader, more variable profiles than in plasmid-mediated editing. We also numerically compared the sizes of deletions and insertions produced by the two delivery methods. RNP-mediated editing tended to yield larger indel sizes across loci, with significant differences (Fig. 4C). RNPs produced larger deletions at the *DAZL* locus (3.60 ± 0.15 bp vs. 2.73 ± 0.26 bp; *P* < 0.05) and the *STRA8* locus (4.81 ± 0.13 bp vs. 3.73 ± 0.04 bp; *P* < 0.01). For *CVH*, RNPs tended to produce larger insertions, with a non-significant but notable difference (0.47 ± 0.06 bp vs. 0.30 ± 0.04 bp; *P* = 0.08). Across all loci, RNP-mediated editing generated more large deletions (≥ 3 bp) than plasmid. Specifically, ≥ 3 bp deletions significantly rose at *DAZL* (30.45% ± 0.87% vs. 20.99% ± 0.74%; *P* < 0.01), *CVH* (78.08% ± 3.03% vs. 60.87% ± 1.06%; *P* < 0.01), and *STRA8* (58.72% ± 0.96% vs. 38.53% ± 0.67%; *P* < 0.0001) with RNP-mediated editing compared with plasmid-mediated editing. Stacked bar graphs further revealed a shift toward larger deletions in RNP-mediated editing (Fig. 4D).

Consistent with observations in LMH cells, RNP-mediated editing in PGCs produced indel profiles similar to those in LMH cells, with large deletion-dominant distributions. However, insertions in PGCs were generally small and less frequent at *DAZL* (0.055 ± 0.05 bp), *CVH* (0.03 ± 0.01 bp), and *STRA8* (0.03 ± 0.01 bp) (Fig. S5).

### RNP-mediated editing shifts DNA repair toward MMEJ in LMH cells

**Fig. 5:**
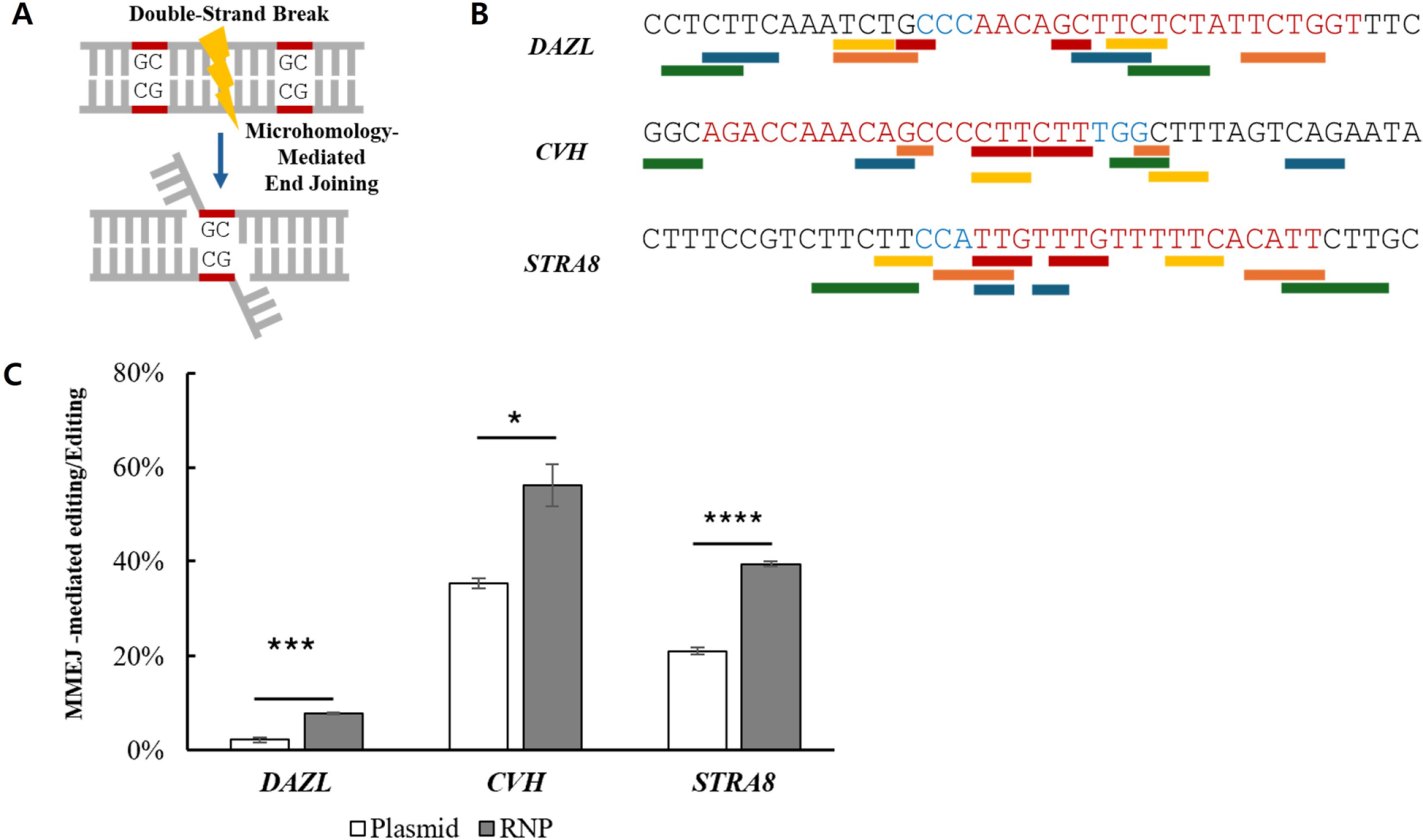
DNA repair analysis with a focus on the MMEJ.** A** Schematic representation of the MMEJ repair pathway following CRISPR/Cas9–induced DSBs. Short microhomology sequences (2–25 bp) flanking the break site are annealed, resulting in characteristic deletion patterns.** B** Five representative examples of microhomology sequences identified at *DAZL*, *CVH*, and *STRA8* loci, with one shared highlighted in the same color.** C** Proportion of MMEJ-associated repair among all detected indel events, representing the contribution of the MMEJ repair pathway to overall genome editing. For all three target sites, RNP-mediated editing resulted in a significantly higher proportion of MMEJ-associated repair compared with the plasmid. Data are presented as mean ± SEM (*n* = 3); Student’s *t*-test; *P < 0.05, ***P < 0.001 and ****P < 0.0001

To explore the potential basis for observed differences in indel size, we analyzed the associated DNA repair pathway, with a specific focus on MMEJ. MMEJ-associated repair is characterized by the use of short microhomology sequences (2–25 bp) flanking double-strand break (DSB) sites, resulting in characteristic deletion events (Fig. 5A). Sequence analysis identified multiple microhomology sequences at the *DAZL*, *CVH*, and *STRA8* loci, with representative examples shown for each target site (Fig. 5B).

Finally, we quantified the contribution of the MMEJ repair pathway by determining the proportion of MMEJ-associated repair among all detected indel events (Fig. 5C). RNP-mediated editing significantly increased the frequency of MMEJ-associated repair at all three loci compared with plasmid at *DAZL* (7.72% ± 0.21% vs. 2.16% ± 0.53%; *P* < 0.001), *CVH* (56.18% ± 4.48% vs. 35.30% ± 1.05%; *P* < 0.05), and *STRA8* (39.41% ± 0.57% vs. 21.11% ± 0.66%; *P* < 0.0001).

Consistent with the observations in LMH cells, RNP-mediated editing in PGCs either produced MMEJ-associated repair at *DAZL* (2.39% ± 0.92%), *CVH* (42.06% ± 1.46%), and *STRA8* (38.90% ± 0.94%) (Fig. S5D).

### Reduced off-target activity with RNP in LMH cells

**Fig. 6:**
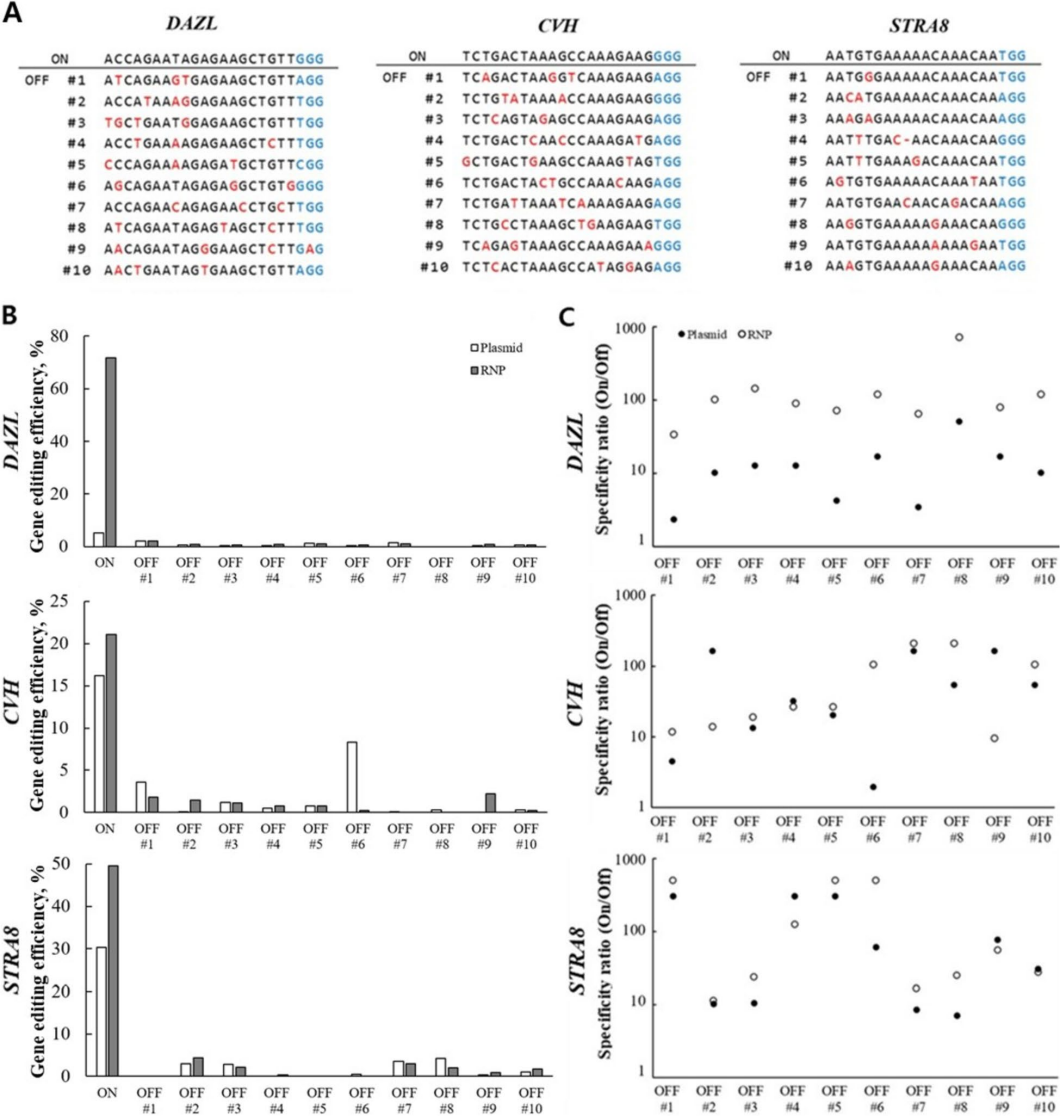
Off-target analysis of RNP- and plasmid-mediated editing in LMH cells. **A** Schematic representation of the on-target site (ON) and the top ten predicted off-target sites (OFF #1–10) for each in *DAZL, CVH*, and *STRA8* gRNA in LMH cells. Nucleotide mismatches relative to the on-target sequence are highlighted in red, whereas black indicates matched bases. PAMs (5′–NGG–3′) are shown in blue. **B** Editing efficiencies at the on-target site and ten predicted off-target sites of each gRNA following editing of either the plasmid or RNP. The editing efficiency at off-target sites was measured using TIDE analysis of Sanger sequencing read data (.ab1). RNP delivery achieved high on-target editing efficiency with minimal detectable off-target editing. **C** Specificity ratios (ON/OFF) calculated for each off-target site under plasmid- and RNP-mediated editing conditions. Overall, RNP delivery consistently exhibited higher specificity than plasmid delivery, indicating more effective suppression of off-target editing

CRISPR/Cas9 RNP delivery is known to minimize off-target editing activity by supporting rapid nuclease activity without prolonged intracellular expression [16]. To evaluate whether delivery method influences the specificity of editing, we predicted off-target (OT) sites for the *DAZL, CVH*, and *STRA8* gRNAs in silico (Fig. 6A; Table S4). The top ten sites were predicted as OT sites for each target site. Indel frequencies at these sites were then quantified and visualized via TIDE analysis (Fig. 6B; Fig. S6) of Sanger sequencing and T7E1 assay (Fig. S4). RNP delivery consistently produced high on-target editing activity with minimal activity at predicted OT sites. In contrast, plasmid-based delivery generated lower on-target efficiencies and showed detectable signals at some off-target sites. Specificity ratios (ON/OFF) calculated for each predicted off-target site further indicated a tendency toward precise on-target editing in RNP-mediated editing (Fig. 6C). RNP delivery exhibited markedly higher specificity across most sites, indicating superior suppression of off-target editing compared with plasmid delivery.

## Discussion

In this study, we quantitatively compared RNP-mediated CRISPR/Cas9 delivery with plasmid-mediated delivery in chicken cells by evaluating key genome editing outcomes, including cell viability, editing efficiency, indel characteristics, and editing specificity. Taken together, our results demonstrate that RNP delivery confers clear advantages in chicken cells.

In both LMH cells and PGCs, RNP delivery maintained cell viability at levels comparable to or higher than plasmid delivery (Figs. 2B and 3B). This is likely because RNPs bypass transcription and translation and persist only transiently in the nucleus, thereby minimizing cellular stress [28]. Consistently higher editing efficiencies across all target sites (Figs. 2C and 3C) further indicate that RNP delivery is an effective and reliable strategy for genome editing in the chicken. Similar outcomes have been reported in mammalian systems [15, 17, 29, 30], suggesting that these observations are not unique to avian systems.

Although RNPs also outperformed plasmids in PGCs, absolute editing efficiencies remained lower than those in LMH cells, suggesting that factors beyond delivery efficiency influence outcomes. Quantitative assessment of plasmid delivery using GFP expression (Fig. S1B and C) indicated approximately a two-fold difference between LMH cells and PGCs, which is insufficient to explain the larger disparity in genome editing efficiency (Figs. 2C and 3C). Moreover, cell type–dependent differences were consistently observed under both plasmid- and RNP-mediated delivery. These findings suggest that, while delivery efficiency contributes to editing outcomes, additional intracellular factors also likely modulate genome editing efficiency. PGCs’ intrinsic cellular factors—such as highly active DNA repair pathways that rapidly resolve DSBs with minimal indel formation, genome-protective responses, and a more compact chromatin architecture—may serve significant roles in modulating editing efficiency [31, 32]. In addition, locus-dependent variation in editing efficiency suggests that local genomic context, including chromatin accessibility and nucleosome positioning, may influence CRISPR/Cas access and cleavage activity [33].

Importantly, while chicken PGCs can be maintained and manipulated in vitro, most avian species lack established PGC lines, limiting the applicability of in vitro genome editing approaches [6]. This limitation has driven interest in developing in vivo genome editing approaches directly in embryos. Because in vivo systems do not allow selection strategies—such as fluorescence- or antibiotic-based enrichment—delivery formats that intrinsically maximize editing efficiency are particularly valuable. In this context, the robust, efficient performance of RNPs observed here supports their potential as an in vivo genome editing modality across avian species.

Beyond improved editing efficiency, RNP delivery in LMH cells generated a markedly broader spectrum of indel patterns (Fig. 4A), with important implications for gene knockout applications. Frameshift mutations arising from indels that are not multiples of three disrupt downstream coding sequences [34] and are often required to achieve robust loss-of-function alleles. Accordingly, increased indel diversity can raise the likelihood of generating frameshifts [35], indicating the suitability of RNP delivery for efficient knockout generation in chicken cells.

RNP delivery also produced significantly larger indels than plasmid delivery at the same loci (Fig. 4C). Large deletions have been linked to increased engagement of MMEJ, which can become favored over classical non-homologous end joining (NHEJ) under certain conditions [36]. The rapid, transient DSBs induced by RNP delivery may similarly promote the use of alternative repair pathways, such as MMEJ. This may explain the increased frequency of larger deletions observed in our data.

Consistent with this interpretation, repair pathway analysis supported that RNP-mediated editing indeed increased the proportion of MMEJ-associated repair across all target loci (Fig. 5C), accompanied by a corresponding increase in ≥ 3 bp deletions (Fig. 4D). These findings suggest that the CRISPR/Cas9 delivery method can influence DNA repair pathway engagement, with RNP-mediated editing favoring MMEJ. Such bias may arise from the high local concentration and transient, yet robust activity of RNP complexes. Notably, MMEJ can yield more predictable deletion patterns driven by defined microhomology sequences, which may be advantageous for applications requiring controlled and predictable genome editing outcomes [25, 37].

Off-target analysis using the T7E1 (Fig. S4B) and Sanger/TIDE (Fig. 6B; Fig. S6) across the top ten predicted off-target sites showed minimal cleavage and indel frequencies in RNP-mediated editing. These results suggest that RNP-mediated editing increases on-target activity without increasing editing activity at major predicted off-target sites. However, given the limited sensitivity of T7E1 and TIDE [38], low-frequency off-target events cannot be assessed. They should be evaluated using high-resolution methods, such as targeted deep sequencing, GUIDE-seq [39], or CIRCLE-seq [40]. Mechanistically, the favorable specificity profile of RNP delivery is plausibly explained by the immediate activity of pre-assembled complexes and their short intracellular persistence, resulting in a rapid burst of editing. In contrast, plasmid delivery requires transcription and translation, leading to delayed and prolonged CRISPR/Cas9 expression, which may increase the cumulative opportunity for off-target events [13, 41].

While our study provides a controlled, head-to-head comparison of plasmid- and RNP-mediated CRISPR/Cas9 delivery in chicken cells, it is important to acknowledge certain limitations. In this study, transfection efficiency for RNP delivery was not independently quantified, and therefore, differences in delivery efficiency may have partially contributed to the observed outcomes. However, as the primary objective was to compare functional editing performance under commonly used and optimized conditions, the results still reflect the combined effects of delivery and intracellular editing activity, which is directly relevant to practical applications. In addition, detailed indel profiling in PGCs after plasmid delivery was not feasible due to consistently low editing efficiencies, which precluded statistically robust characterization of indel spectra. Notably, this limitation underscores the technical challenges of plasmid-mediated editing in PGCs and further highlights the advantages of RNP-based approaches for germline-competent cells. Additionally, we did not include viral vectors, such as lentivirus and avian adeno-associated virus (A3V), or advanced delivery modalities, such as lipid nanoparticles (LNPs) or cell-penetrating peptides (CPPs). Although these systems can achieve high delivery efficiencies, they introduce additional variables—including vector production, cell-type tropism, and genomic integration—that differ fundamentally from transient liposome-mediated transfection [42–46]. Incorporating such approaches would expand the scope beyond the intended focus on intrinsic differences between CRISPR delivery formats. Finally, this work is limited to in vitro analyses. Evaluation of in vivo delivery and germline transmission is a critical next step to establish the practical utility of RNP-mediated genome editing in chickens. Future studies should systematically compare RNP delivery with viral and other advanced platforms, optimize delivery strategies for PGCs, and assess editing efficiency and germline transmission in vivo. By explicitly defining these limitations and outlining directions for future research, we clarify the study’s scope while highlighting the mechanistic insights from our controlled comparison of CRISPR formats.

## Conclusions

In this study, we demonstrate that RNP-based CRISPR/Cas9 delivery provides a robust and efficient genome editing strategy in chicken cells, including PGCs. Compared with plasmid delivery, RNP delivery consistently achieved higher editing efficiencies while maintaining comparable or improved cell viability and markedly reducing off-target editing activity. Importantly, RNP-mediated editing produced broader and larger indel profiles, accompanied by a pronounced shift toward MMEJ. This repair pathway bias highlights the influence of CRISPR/Cas9 delivery format on DNA repair outcomes. It suggests that RNP delivery may be particularly useful for applications that require efficient gene disruption or predictable deletion patterns. Although this study was conducted under in vitro conditions, the superior performance of RNP delivery observed here underscores its potential utility for in vivo genome engineering, especially in species lacking established PGC culture systems. Together, these findings position RNP-mediated CRISPR/Cas9 delivery as a flexible and reliable strategy for chicken genetic modification, providing a strong foundation for advancing PGC-based and embryo-based genome editing technologies.

## Supplementary Information

Additional file 1: Table S1. Oligonucleotides used for plasmid construction and sgRNA IVT. Table S2. PCR primer sets and amplification parameters. Table S3. In silico predicted MMEJ-associated indel patterns. Table S4. In silico predicted off-target sites. Fig. S1. (A) Schematic representation of the Cas9 expression plasmids (PX459 and PX458). (B) Quantification of GFP-positive cells over time following PX458 plasmid transfection in LMH cells and PGCs. The percentage of GFP-positive cells was determined at the indicated time points. (C) Representative flow cytometry histograms showing GFP fluorescence intensity at each indicated time point in LMH cells and PGCs. Fig. S2. Quality of the in vitro transcribed (IVT) single guide RNAs (sgRNAs). Fig. S3. Preliminary optimization of RNP transfection conditions. Fig. S4. T7E1 assay for on- and off-target sites of RNP- and plasmid-mediated editing in LMH cells. Fig. S5. Characterization of indel profiles generated by RNP-mediated editing in PGCs. Fig. S6. Sanger sequencing read data for off-target sites of RNP- and plasmid-mediated editing in LMH cells.

## Data Availability

All data generated or analyzed during this study are included in this published article and its supplementary information files.
