# Supplementary material for: Improvement of chicken genome editing efficiency in vitro using ribonucleoprotein-mediated CRISPR/Cas9 delivery
Source: J Anim Sci Biotechnol. 2026 May 4;17:82. doi: 10.1186/s40104-026-01402-1 (PMC13137644; doi:10.1186/s40104-026-01402-1)
Supplement: Supplementary file 1 — Additional file 1: Table S1. Oligonucleotides used for plasmid construction and sgRNA IVT. Table S2. PCR primer sets and amplification parameters. Table S3. In silico predicted MMEJ-associated indel patterns. Table S4. In silico predicted off-target sites. Fig. S1. (A) Schematic representation of the Cas9 expression plasmids (PX459 and PX458). (B) Quantification of GFP-positive cells over time following PX458 plasmid transfection in LMH cells and PGCs. The percentage of GFP-positive cells was determined at the indicated time points. (C) Representative flow cytometry histograms showing GFP fluorescence intensity at each indicated time point in LMH cells and PGCs. Fig. S2. Quality of the in vitro transcribed (IVT) single guide RNAs (sgRNAs). Fig. S3. Preliminary optimization of RNP transfection conditions. Fig. S4. T7E1 assay for on- and off-target sites of RNP- and plasmid-mediated editing in LMH cells. Fig. S5. Characterization of indel profiles generated by RNP-mediated editing in PGCs. Fig. S6. Sanger sequencing read data for off-target sites of RNP- and plasmid-mediated editing in LMH cells. [file 40104_2026_1402_MOESM1_ESM.pdf]

SUPPLEMENTARY TABLE

Table S1. Oligonucleotides used for plasmid construction and sgRNA IVT

Name	5'–Oligo Seq–3'	Usage
DAZL PX459	F: CAC CGA CCA GAA TAG AGA AGC TGT T<br>R: AAA CAA CAG CTT CTC TAT TCT GGT C	PX459 golden gate
CVH PX459	F: CAC CGT CTG ACT AAA GCC AAA GAA G<br>R: AAA CCT TCT TTG GCT TTA GTC AGA C	
STRA8 PX459	F: CAC CGA ATG TGA AAA ACA AAC AA<br>R: AAA CTT GTT TGT TTT TCA CAT TC	
DAZL IVT	F: TAA TAC GAC TCA CTA TAG ACC AGA ATA GAG AAG C<br>R: TTC TAG CTC TAA AAC AAC AGC TTC TCT ATT CTG G	sgRNA IVT
CVH IVT	F: TAA TAC GAC TCA CTA TAG TCT GAC TAA AGC CAA A<br>R: TTC TAG CTC TAA AAC CTT CTT TGG CTT TAG TCA G	
STRA8 IVT	F: TAA TAC GAC TCA CTA TAG AAT GTG AAA AAC AA<br>R: TTC TAG CTC TAA AAC TTG TTT GTT TTT CAC AT	

Table S2. PCR primer sets and amplification parameters

Name	5'–Oligo Seq–3'	Amplicon Size (bp)	Annealing Temp. (°C)	Usage
DAZL NGS	F: TGT GCC CTC CTC TCC TGG AA<br>R: CCT CAG GAG CAA TCT GCT GAA AGG T	500	60	NGS
CVH NGS	F: TTG CTT GGC CTG AAG GTA AC<br>R: CCT TGG TCG GCC ACC TTT AT	298	57	
STRA8 NGS	F: TGA TAC CAC ACT GGG ATT CC<br>R: GTA TTT CCT TTG CTT CCT ATG C	513	60	
DAZL OT #1	F: GGC CCT TGA CAT TTT GCC AG<br>R: TCC TGC TTG GGT GAA TGA GC	352	60	Off-Target
DAZL OT #2	F: AGG GTT AAG AGA GGA GTG TCA A<br>R: CCA AGT ACA ATC CCA GTG GCT	371	60	
DAZL OT #3	F: GAG GTG ATG GGT GGA GCT TC<br>R: TGC CTT TCA GGT CAG AGC AG	358	60	
DAZL OT #4	F: TTG CAC GGA TCT TGG TTA TGG<br>R: CCA TGT CCA GGT GGC TTT TG	332	60	
DAZL OT #5	F: TTG GTC TTG TGT GGC AAA GC<br>R: GCC TGT CCC ATT TTG TGC AG	349	60	
DAZL OT #6	F: TGT GAT GCT ACA GCC TGA AGG<br>R: AGC TCT TCA AAC CAC TGG CA	383	60	
DAZL OT #7	F: GGG ACC AGA AAG CAG CCA A<br>R: TCT CCT GTC GTT TCT GAG GC	320	60	
DAZL OT #8	F: ACA CTC GTT TCG AAA CAC TTC C<br>R: ATT GAA GGC CAG GCT GGA TG	375	60	
DAZL OT #9	F: CAC TTA CTA TGC ATT CTG GGT GT<br>R: CAG GAG ACC AGA ACA GTG AGG	392	57	
DAZL OT #10	F: CTT GAG CTG TGA GGG ATC GG<br>R: CAT TGC ATG CAC CTG AGC TC	360	60	
CVH OT #1	F: AGC ATG CCT CAC TGA GCA G<br>R: AGA GGT GCA CAG AAG GAA GC	335	60	
CVH OT #2	F: GAC CTT TCC AGC TCT AGT GCA<br>R: GGT ACC AAC CAA CCA CTT GT	358	60	
CVH OT #3	F: AGC ACA TGG GGA CTC AGT TG<br>R: TGT CTT GGC CAA GAG TGT CC	378	60	
CVH OT #4	F: GCT GGC TTT CTT GTC CAC AG<br>R: ATT GAA TGG CCC AAC ACT CA	293	60	
CVH OT #5	F: GGA AGA TCA AGC AAG GGC CA<br>R: CCA AGG AGG AAA GCA GAG CA	374	60	
CVH OT #6	F: GCA GAG TGC TTC AGT CCA GA<br>R: CCA AGG AGG AAA GCA GAG CA	335	60	
CVH OT #7	F: ACA AGT AAT CGT GCG TGC AC<br>R: ACG GAA AGA GCG GTG ATA CG	393	60	
CVH OT #8	F: CGC CTG TGT AAT CAT TGC CTC<br>R: GAA GGC AGC AAC TTC TGT GG	362	57	
CVH OT #9	F: CAT TGG TCA CAT TGC CGC TC<br>R: AGT TTC CTG CTG TGC CCT AC	366	60	
CVH OT #10	F: GGG GTT TGC TGG AGT GTG ATA<br>R: CAT TGC TCA CTG CTT GCC TC	350	60	

STRA8 OT #1	F: TGG CAA AGG CTT TAT ATT GAT CC<br>R: TTG TTT GTG GCT GGC AGA CA	376	60
STRA8 OT #2	F: GCC AGG AGC ATC ATT CCT TC<br>R: TGG AGC AGA CCT TGT GCA TT	358	60
STRA8 OT #3	F: GGT TTT GCA CTG ATG GCT GG<br>R: AAG GAT ATG GAG GGG CCC TT	316	60
STRA8 OT #4	F: AGT GCA TTT GAA CTG TGT TCA<br>R: CAC AGT TGG AAT GTG CGC TC	387	57
STRA8 OT #5	F: CGT TAC CTG GGA GAA GAG GC<br>R: GCT GTG ACA CGA AGC CCT AT	372	63
STRA8 OT #6	F: TCC TGC ATG AGA AGA AGG CC<br>R: GGG TCT GCC TGT TCC TTT CA	300	60
STRA8 OT #7	F: TGG AAG TGT TCA AGG CCA GG<br>R: GTC CTC ATG CTC AGT GCC TC	384	60
STRA8 OT #8	F: GAG CCT TTT TGG AAA ACG GGA<br>R: GTG TAG CCA CCT CCA CTG AC	374	60
STRA8 OT #9	F: GAT ACT GCT TTC GGC GCA<br>R: AGA CCA GAC TGC AAT ACA CTG	322	60
STRA8 OT #10	F: CAG AGC ACA GGT ACA GGG C<br>R: GTA AGT TGT GTT CTG CTG CGG	301	60

Table S3. In silico predicted MMEJ-associated indel patterns

	Predicted Patterns	Microhomology	Deletion Length
	CCTCTTCAAATCTGC--------TTCTCTATTCTGGTTTC	GC	8
	CCTCTTC-------------------TCTATTCTGGTTTC	CTTC	19
	CCTCTTCAAATCTG---------------------GTTTC	TCTG	21
	CCTCTTCAAATCT--------------CTATTCTGGTTTC	TCT	14
	CCTCT------------------------ATTCTGGTTTC	CTCT	24
	CCTCTTCAAATCT----------------ATTCTGGTTTC	TCT	16
	CCTCTTCAAATCT-----------TCTCTATTCTGGTTTC	CT	11
	CCTCT----------------------CTATTCTGGTTTC	TCT	22
	CCTCTTC--------------------------TGGTTTC	TTC	26
DAZL	CCT---------------------TCTCTATTCTGGTTTC	CT	21
	CCTCTTC---------------------TATTCTGGTTTC	TC	21
	CCTCT-----------------------------GGTTTC	TCT	29
	CCT--------------------------ATTCTGGTTTC	CT	26
	CCTCTTC---------------------------------	TTC	33
	CCTCTTCAAATC----------------------------	TC	28
	CCTCTTCAAAT--------------------TCTGGTTTC	AT	20
	CCT-------------------------------GGTTTC	CT	31
	CCTC------------------------------------	TC	36
	CCTCTT--------------------------------TC	TT	32
	GGCAGACCAAACAGCCCCTT---TGGCTTTAGTCAGAATA	CTT	3
	GGCAGACCAAACAGCCCCTT---------TAGTCAGAATA	CTT	9
	GGCAGACCAAACAGC------------TTTAGTCAGAATA	GC	12
	GGC------------------------TTTAGTCAGAATA	GGC	24
	GGCAGACCAAACAG----------------------AATA	CAG	22
	GGCAGACCAAACAGCCCCTT----GGCTTTAGTCAGAATA	TT	4
CVH	GGCAGA-------------------------------ATA	CAGA	31
	GGCAGACCAAACAG------------------TCAGAATA	AG	18
	GGCAGACCAAACAGCCCCTT----------AGTCAGAATA	TT	10
	GGCAGACCA--------------------------GAATA	CA	26
	GGCAG---------------------------TCAGAATA	AG	27
	GGCAGACCAAA---------------------------TA	AA	27
	GGCAGACCAA----------------------------TA	AA	28
	CTTTCCGTCTTCTTCCATTG----TTTTCACATTCTTGC	TTG	4
STRA8	CTTTCCGTCTTCTTCCATT----------------CTTGC	CATT	16
	CTTTCCGTCTTCTTC---------------ACATTCTTGC	TTC	15

CTTTCCGTCTTCTT------------------------GC	TTCTT	24
CTTTCCGTCTTCTTCCATT---TGTTTTTCACATTCTTGC	TT	3
CTTTCCGTCTTC------------------ACATTCTTGC	TTC	18
CTTTCCGTCTTCTTCCATTG-------------------C	TTG	19
CTTTCCGTCTTCTTCCA--------------CATTCTTGC	CA	14
CTTTC-------------------------ACATTCTTGC	TTTC	25
CTTTCCGTCTTCTTCCATT-------TTTCACATTCTTGC	TT	7
CTTTCCGTCTTCTTC---------------------TTGC	TTC	21
CTTTCCGTCTTCTT--------TGTTTTTCACATTCTTGC	TT	8
CTTTCCGTCTTCTTCCATT--------TTCACATTCTTGC	TT	8
CTTTCCGTCTT---------------------------GC	TCTT	27
CTTTCCGT-----------------TTTTCACATTCTTGC	GT	17
CTTTCCGTCTTCTT---------GTTTTTCACATTCTTGC	TT	9
CTTTCCGTCTTCTTCCATT---------TCACATTCTTGC	TT	9
CTTTCCGTCTTCTTCCATT----------CACATTCTTGC	TT	10
CTTT-------------------GTTTTTCACATTCTTGC	TTT	19
CTTTCCGTCTT-----------TGTTTTTCACATTCTTGC	TT	11
CTTTCCGTCTT------------GTTTTTCACATTCTTGC	TT	12
CTTTCCGTCTTCTT------------TTTCACATTCTTGC	TT	12
CTTTCCGTC---------------------ACATTCTTGC	TC	21
CTTTCCGTCTTCTT-------------TTCACATTCTTGC	TT	13
CTTTCCGTCTTCTT--------------TCACATTCTTGC	TT	14
CTTT-----------------------TTCACATTCTTGC	TTT	23
CTTTCCGTCTT---------------TTTCACATTCTTGC	TT	15
CTTT------------------------TCACATTCTTGC	TTT	24
CTTTCCGTCTT----------------TTCACATTCTTGC	TT	16
CTTTCCGTCTT-----------------TCACATTCTTGC	TT	17
CTTTC-------------------------------TTGC	TTC	31
CTTT------------------TGTTTTTCACATTCTTGC	TT	18
CTT--------------------GTTTTTCACATTCTTGC	TT	20
CTT-----------------------------------GC	CTT	35
CTTT----------------------TTTCACATTCTTGC	TT	22
CTT--------------------------CACATTCTTGC	TT	26
CTT--------------------------------CTTGC	TT	32
CTTT----------------------------------GC	TT	34

Table S4. In Silico Predicted Off-Target Sites

Name	5' –Off Target Seq–3'	Chromosome	Locus	Mismatch
DAZL OT #1	AtC AGA Agt GAG AAG CTG TT	CM000095.5	62414982	3
DAZL OT #2	ACC AtA Aag GAG AAG CTG TT	CM000095.5	98311727	3
DAZL OT #3	tgC tGA ATg GAG AAG CTG TT	CM000115.5	2551761	4
DAZL OT #4	ACC tGA AaA GAG AAG CTc TT	CM000093.5	173751908	3
DAZL OT #5	cCC AGA AaA GAG AtG CTG TT	CM000095.5	88212792	3
DAZL OT #6	AgC AGA ATA GAG AgG CTG Tg	CM000094.5	98729833	3
DAZL OT #7	ACC AGA AcA GAG AAc CTG cT	CM000096.5	9436440	3
DAZL OT #8	AtC AGA ATA GAG tAG CTc TT	CM000096.5	29643339	3
DAZL OT #9	AaC AGA ATA GgG AAG CTc TT	CM000098.5	34626816	3
DAZL OT #10	AaC tGA ATA GtG AAG CTG TT	CM000107.5	11863837	3
CVH OT #1	TCa GAC TAA gGt CAA AGA AG	CM000099.5	31449905	3
CVH OT #2	TCT Gta TAA AaC CAA AGA AG	CM000097.5	19789872	3
CVH OT #3	TCT cAg Tag AGC CAA AGA AG	CM000123.5	5273876	3
CVH OT #4	TCT GAC TcA AcC CAA AGA ta	CM000094.5	37234538	4
CVH OT #5	gCT GAC TgA AGC CAA AGt AG	CM000094.5	96618610	3
CVH OT #6	TCT GAC Tac tGC CAA AcA AG	CM000096.5	2617052	3
CVH OT #7	TCT GAt TAA AtC aAA AGA AG	CM000098.5	27095198	3
CVH OT #8	TCT GcC TAA AGC tgA AGA AG	CM000099.5	242336	3
CVH OT #9	TCa GAg TAA AGC CAA AGA Aa	CM000100.5	3028764	3
CVH OT #10	TCT cAC TAA AGC CAt AGg AG	CM000119.5	2899381	3
STRA8 OT #1	AAT GgG AAA AAC AAA CAA	CM000100.5	1581931	1
STRA8 OT #2	AAc aTG AAA AAC AAA CAA	CM000096.5	26313749	2
STRA8 OT #3	AAa GaG AAA AAC AAA CAA	CM000093.5	68336187	2
STRA8 OT #4	AAT tTG Ac- AAC AAA CAA	CM000093.5	147880695	3
STRA8 OT #5	AAT tTG AAA gAC AAA CAA	CM000093.5	59268968	2
STRA8 OT #6	AgT GTG gAA AAC AAA tAA	CM000094.5	54274633	3
STRA8 OT #7	AAT GTG AAc AAC AgA CAA	CM000095.5	107184817	2
STRA8 OT #8	AAg GTG AAA AAg AAA CAA	CM000096.5	8703396	2
STRA8 OT #9	AAT GTG AAA AAa AAA gAA	CM000096.5	83138770	2
STRA8 OT #10	AAa GTG AAA AAg AAA CAA	CM000107.5	7833365	2

SUPPLEMENTARY FIGURE

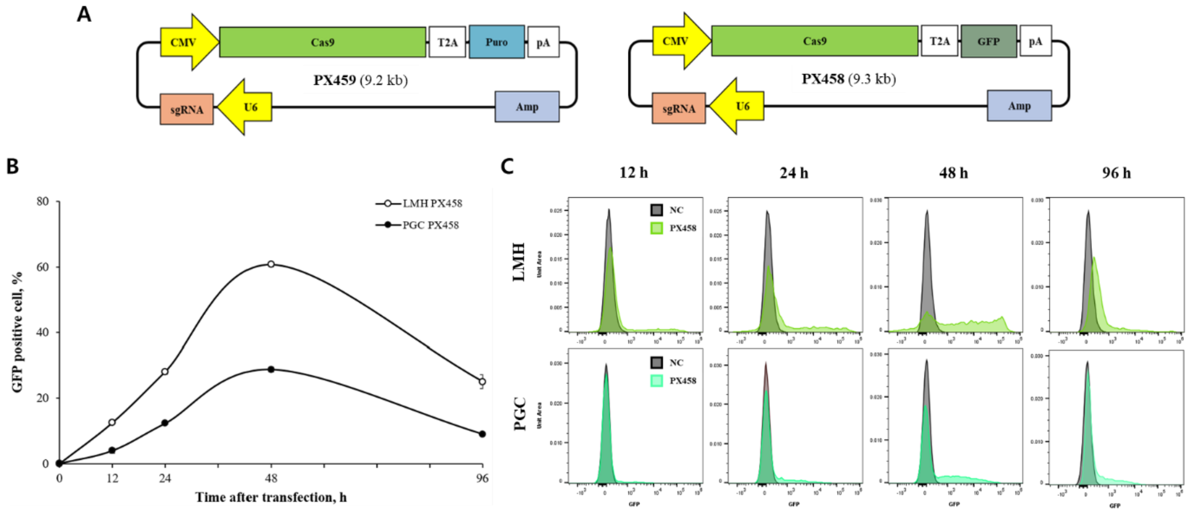

Fig. S1. Characterization of plasmid expression in LMH cells and PGCs (A) Schematic representation of the Cas9 expression plasmids (PX459 and PX458). **(B)** Quantification of GFP-positive cells over time following PX458 plasmid transfection in LMH cells and PGCs. The percentage of GFP-positive cells was determined at the indicated time points. **(C)** Representative flow cytometry histograms showing GFP fluorescence intensity at each indicated time point in LMH cells and PGCs.

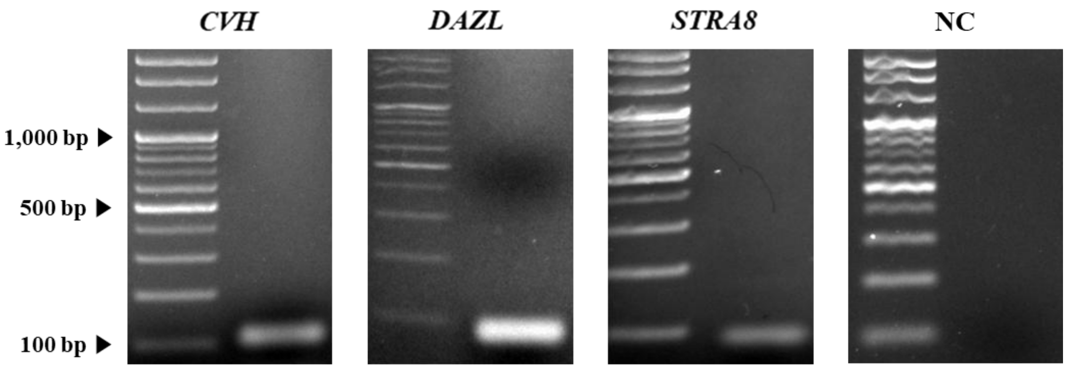

Fig. S2. Quality of IVT sgRNAs IVT sgRNAs were assessed by electrophoresis on a 2% agarose gel and were correctly synthesized with the expected size of approximately 100 bp—negative control; IVT without transcriptase.

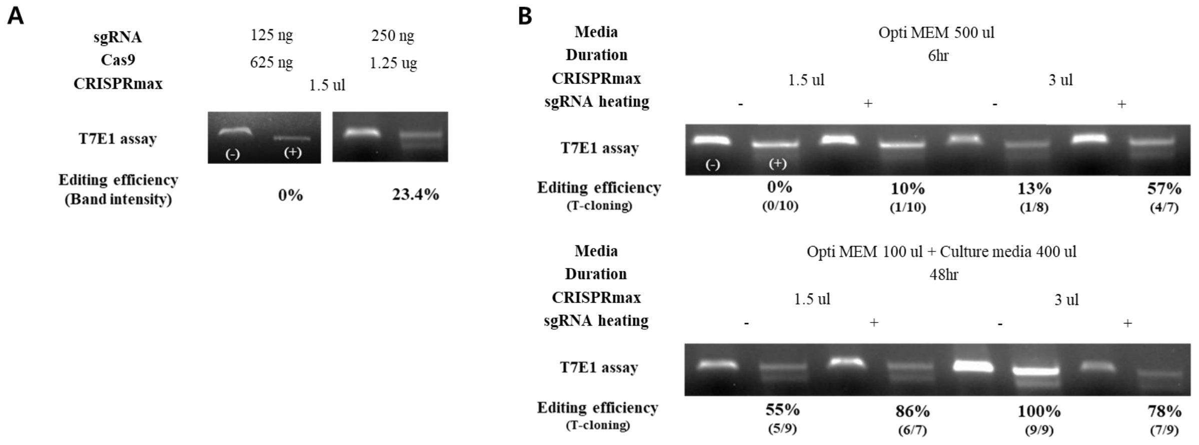

Fig. S3. Preliminary optimization of RNP transfection conditions (A) Determination of optimal RNP quantity using T7E1 band intensity assay. **(B)** Optimization of RNP complex formation and transfection parameters using T7E1 assay and T-cloning.

A

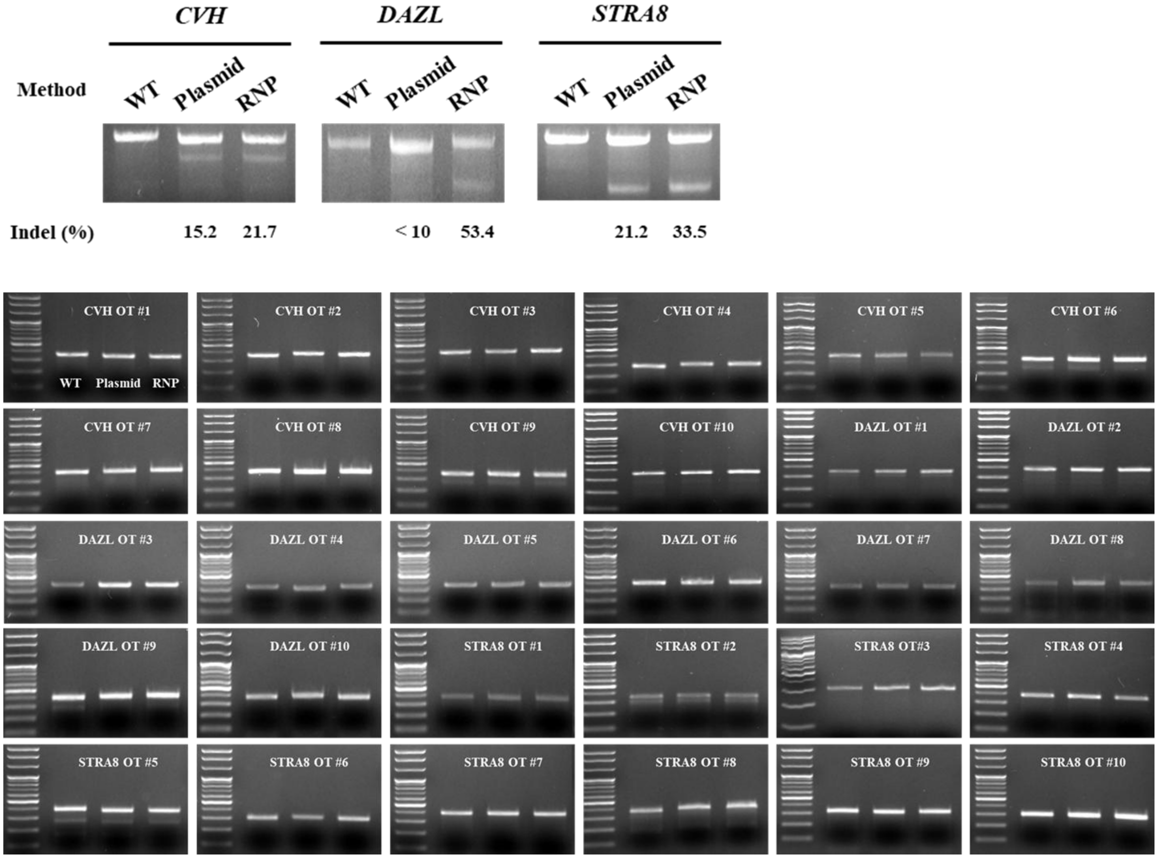

Fig. S4. T7E1 assay for on- and off-target sites of RNP- and plasmid-mediated editing in LMH cells (A) T7E1 assay of the on-target amplicons for each treatment group, with WT, plasmid-, and RNP-mediated edited samples loaded in the indicated order. Cleaved DNA fragments generated by the mismatch-sensitive T7E1 assay indicate the presence of indels at the target site, showing an editing efficiency trend comparable to that observed in NGS-based analysis. **(B)** T7E1 assay results for predicted off-target (OT) sites associated with *DAZL*, *CVH*, and *STRA8* gRNAs (OT #1–10). For each off-target site, WT, plasmid-, and RNP-mediated edited samples were analyzed to detect potential unintended genome modifications. No bands distinguishable from the WT control were detected at any of the predicted sites. DNA ladders are shown where applicable.

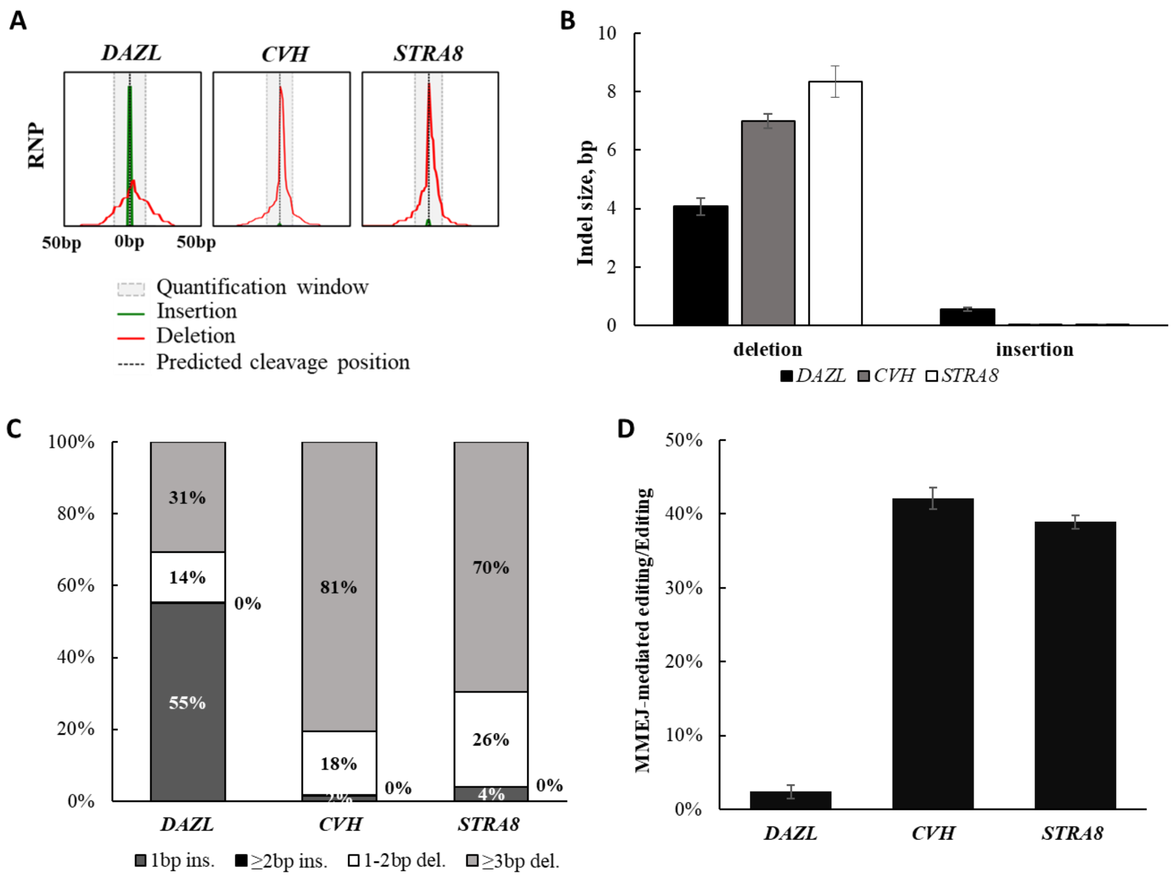

Fig. S5. Characterization of indel profiles generated by RNP-mediated editing in PGCs (A) Indel position distribution plots. Red peaks represent deletion events and green peaks indicate insertion events. The vertical dashed lines mark the predicted cleavage positions, and the grey-shaded region denotes the quantification window used for analysis. **(B)** Indel sizes of RNP-mediated editing. RNP-mediated editing showed a trend toward generating large deletions and small insertions (bp) in *DAZL, CVH,* and *STRA8*. **(C)** Stacked bar graphs showing the relative proportions of each indel category. Distribution of indel types among total genome editing events, categorized by indel size. For all three target sites. **(D)** Proportion of MMEJ-associated repair among all detected indel events, representing the contribution of the MMEJ repair pathway to genome editing. Data are presented as mean ± SEM (n = 3).

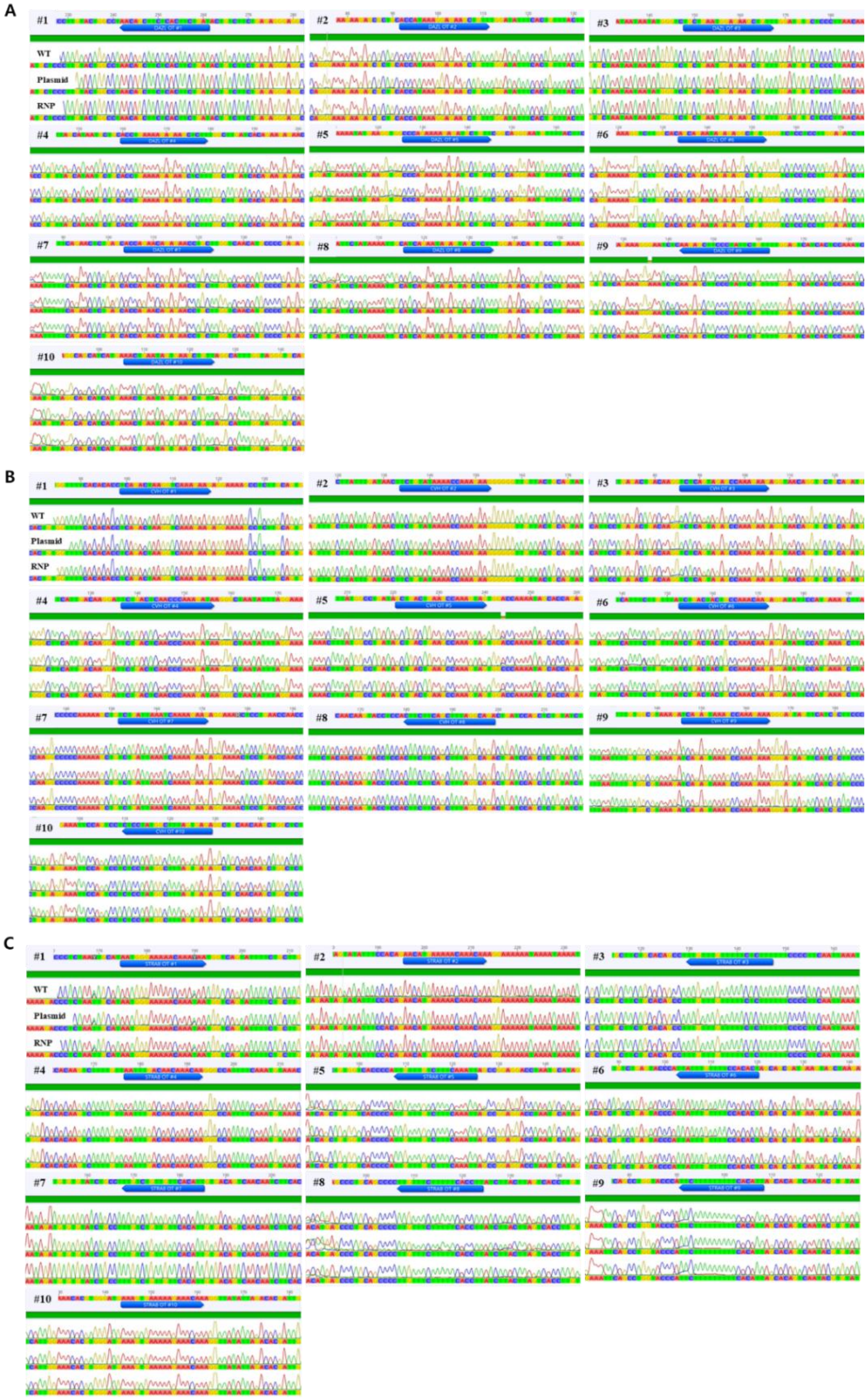
A
#1
#2
#3
WT
Plasmid
RNP
#4
#5
#6
#7
#8
#9
#10
B
#1
#2
#3
WT
Plasmid
RNP
#4
#5
#6
#7
#8
#9
#10
C
#1
#2
#3
WT
Plasmid
RNP
#4
#5
#6
#7
#8
#9
#10

Fig. S6. Sanger sequencing read data for off-target sites of RNP- and Plasmid-mediated editing in LMH cells (A-C) Sanger sequencing read data of the ten predicted OT sites associated with *DAZL* (**A**), *CVH* (**B**), and *STRA8* (**C**) guide RNAs (gRNAs) (OT #1–10). For each off-target site, WT, plasmid-, and RNP-mediated edited samples were shown. Minimal alterations in the peak profiles distinguishable from the WT control were detected at any of the tested sites.
